# Computational Resolution Enhancement of Mitochondria monitoring in Multiple Organs using Intravital Two-Photon Microscopy

**DOI:** 10.7150/ijms.123395

**Published:** 2026-01-01

**Authors:** Saeed Bohlooli Darian, Jeongmin Oh, Jun Ki Kim

**Affiliations:** 1 Department of Biomedical Engineering, College of Medicine, University of Ulsan College of Medicine, Seoul 05505, Republic of Korea.; 2 Biomedical Engineering Research Center, Asan Institute for Life Sciences, Asan Medical Center, Seoul, 05505, Republic of Korea.

**Keywords:** two-photon intravital microscopy, image processing, eSRRF, denoising, mitochondria, super-resolution

## Abstract

**Background and Objective:** Understanding living cell mechanisms and enabling intracellular monitoring requires advanced and often costly imaging technologies. Conventional fluorescence microscopy is widely used but suffers from resolution limitations, making it challenging to capture fine subcellular structures like mitochondria. While super-resolution microscopy may overcome these constraints, it introduces tradeoffs, including its limitation in intravital imaging fields and complexity in analyzing multiple cells simultaneously. To address these challenges, we developed a computational approach that enhances resolution and signal clarity without the need for specialized hardware, applicable for intravital imaging studies.

**Methods:** We utilized Dendra2 transgenic mice to observe mitochondria in hepatocytes under normal physiological condition and in response to alcohol-induced liver stress. To achieve high-resolution *in vivo* imaging, we employed high-magnification objectives, ensuring precise visualization of subcellular structures. We implemented a trained self-supervised denoising model to suppress background noise and improve signal intensity, ensuring a clearer visualization of the mitochondria in hepatic cells. Additionally, enhanced super-resolution radial fluctuations (eSRRF) analysis was applied to image sequences to achieve subcellular resolution. Various numerical modifications and parameter optimizations were performed to refine the technique. The methodology was validated through computational analysis of different imaging conditions to assess its robustness and effectiveness.

**Results:** Our approach successfully enhanced image resolution to the subcellular level, enabling the visualization of discrete mitochondrial structures and monitoring intracellular events *in vivo*. The denoising model effectively reduced background interference while preserving essential biological signals. Furthermore, the application of eSRRF significantly improved the clarity of cellular components, even when the original images exhibited poor lateral resolution, allowing for improved interpretation of intracellular structures.

**Conclusions:** The proposed computational technique provides a cost-effective and accessible solution for achieving super-resolution live imaging without the need for high-end microscopy equipment. By reducing noise and enhancing resolution, this approach facilitates detailed intracellular analysis, suitable for live animal studies.

## 1. Introduction

Fluorescence microscopy has transformed the cell biology by enabling the study of molecular and organelle monitoring in living cells. However, its resolution is limited by the optical diffraction 1, 2, restricting observations to structures larger than half the wavelength of light. While electron 3 and super-resolution 4-6 microscopy overcome this limitation, they are unsuitable for live animal studies and require extensive, time-intensive sample preparation 7, and often destructive fixation procedures 8-10. These limitations highlight the need for alternative high-resolution imaging methods that are more versatile and minimally invasive.

Meanwhile, appropriate selection of microscopy technique 11 is critical for achieving optimal image resolution. Each method offers distinct advantages depending on penetration depth, resolution, and sample properties. Multi-photon microscopy 12, 13 stands out for its use of longer wavelengths, reducing photodamage and photobleaching while enabling imaging of deeper layers 14. This makes it particularly effective for overcoming light-scattering limitations in traditional methods.

Advancements in computational imaging and artificial intelligence have significantly improved analytical methods for enhancing resolution by increasing local contrast, reducing background noise, and improving feature distinguishability. Advanced self-supervised denoising algorithms 15, allow noise reduction without requiring paired clean datasets, enabling practical deployment in intravital experiments. Meanwhile, the enhanced super-resolution radial fluctuations (eSRRF) algorithm 16, 17 offers a powerful yet simple approach to further improve the resolution. By analyzing temporal fluctuations across image sequences, eSRRF extracts subpixel information, reconstructing finer details that remain unresolved in raw data. Two key parameters optimize eSRRF performance: the ring radius (RR), which defines the surrounding subpixels included in the analysis, and Sensitivity, which sets the threshold for detecting local intensity fluctuations to balance resolution enhancement with noise amplification. By improving image quality and the resolution, eSRRF provides additional visual details, expanding the scope of insights that can be derived from experimental data.

Conventional microscopy methods 18 often struggle to accurately capture the positions and morphologies of small organelles due to their complexity and acquisition time. Mitochondria 19-21, known as the "powerhouses of the cell", are critical organelles involved in energy production and essential metabolic processes. These double-membraned structures retain their own genetic material and machinery for generating adenosine triphosphate (ATP) 22 through oxidative phosphorylation. Mitochondrial dysfunction has been implicated in various human diseases 23, 24, making their study a topic of growing scientific interest. In particular, the effects of ethanol (EtOH) consumption on cellular structures, including hepatic mitochondria 25, have drawn significant attention due to their relevance to metabolism and disease. Despite their critical role in human physiology and disease, the study of mitochondrial morphology and dynamics remains limited by current imaging resolution 26. Enhanced resolution could unlock new insights and drive further progress in mitochondrial research.

In this study, we developed a computational method integrating a conventional two-photon microscope with a trained denoising model, Gaussian filtering 27, and eSRRF to enhance resolution beyond the sub-cellular level in various organs imaging. To validate the resolution enhancement, we analyzed mitochondrial morphology under normal conditions and in a simulated alcoholic liver disease (ALD) mouse model. This included examining mitochondrial alterations due to EtOH exposure 28, 29 and lipid accumulation in hepatocytes 30. By demonstrating the method's effectiveness, we aim to advance biomedical imaging techniques and provide practical insights to achieve high-resolution images in diverse *in vivo* studies.

## 2. Method

### 2.1. Animal experiments

Dendra2 mitochondria-tagged mice were obtained from the Jackson Laboratory (#018397, USA) and a local matrilineal colony was maintained ensuring their suitability for our experimental needs. Mice were weaned at 4 weeks of age and raised sex-segregated at five animals per cage, under a 12h light:dark cycle at 23°C, with food and water available ad libitum. Mice weighing between 20-30g were used in our experiment. Anesthetic solution was prepared by diluting mixture of 6% v/v Zoletil, 4% v/v Rompun (Xylazine) in PBS solution, and it was administrated intraperitoneally. Following anesthesia, the chest fur was shaved to facilitate imaging, and mice were placed on a temperature-controlled microscope stage to maintain stable body temperature during imaging session. The imaging initiated with the inspection of abdominal skin for mitochondria imaging, followed by the observation of the liver and various organs such as the kidney, muscle, and heart for comprehensive imaging analysis.

All animal experiments followed the guidelines of the Korean Ministry of Food and Drug Safety under the Laboratory Animal Act and were approved by the Institutional Animal Care and Use Committee of the Asan Institute for Life Sciences (2021-12-030).

### 2.2. Imaging setup

Intravital imaging was performed using a two-photon microscope (IVM-MS, IVIM Technology, Korea) equipped with a Ti:Sapphire laser tuned to an excitation wavelength of 920 nm. Enabling precise acquisition of specific fluorescent channels, the microscope was equipped with a broad emission range of 185 to 760 nm. High-resolution imaging was achieved using a high-performance objective lens (100×, NA 1.45, Olympus, Japan).

### 2.3. Image analysis

Time-lapse image sequences were captured and processed using Fiji software 31 to optimize outcomes. To mitigate the impact of motion artifacts, image registration was applied to align frames and eliminate drift 32. To attenuate background noise, we employed the DenoiSeg plugin in Fiji, as described in Buchholz et al. 33, which integrates self-supervised denoising based on Noise2Void (N2V) framework with optional structural priors for segmentation. In this approach, the model learns to predict masked pixels from their surrounding context, eliminating the need for clean ground-truth images. The network architecture consists of a U-Net with four down-sampling and up-sampling layers connected via skip connections, preserving fine structural details. For structural guidance, 2-10 manually segmented images generated with Labkit 34 were incorporated, allowing the network to better preserve key mitochondrial features. Training was performed using a masked mean squared error (MSE), loss applied only to masked pixels with the dataset split into ~85% for training and ~15% for validation, optimized over multiple epochs until convergence. This strategy provided robust denoising performance while consistently maintaining mitochondrial morphology, without introducing artificial structures, and yielded reproducible results across repeated training runs. Alternatively, a Gaussian blur filter was applied to the image for subsequent processing and to provide a baseline for comparison with the denoising model.

For resolution enhancement, the eSRRF algorithm was applied to both denoised and Gaussian-filtered images. By leveraging subpixel correlations in time-series data, eSRRF improves representation of fine structures. Key eSRRF parameters were systematically optimized using the Quality and Resolution (QnR) score as a quantitative metric of reconstruction quality. Parameter ranges were defined in the eSRRF parameter-sweep application by specifying a start value and step size. The resulting QnR map allows to identify conditions that minimizes artifacts (e.g., patterning, over-sharpening, or low-resolution reconstructions) while maximizing the QnR score.

To assess the efficiency of the proposed methodology, and to conduct a thorough comparative analysis between the resolution values of raw images and their corresponding enhanced counterparts, we employed rolling Fourier Ring Correlation (rFRC) analysis 35, implemented within the Fiji platform. This robust method provided an objective metric for comparing the spatial resolution of raw and enhanced images, enabling thorough validation of the proposed image processing pipeline.

### 2.4. Alcoholic liver and lipid droplets detection

To establish a model of ALD, mice received oral gavage of ethanol (EtOH) at a dose of 6 g/kg 36. Two groups of six mice (equal gender ratio) were administered three doses of PBS (control) or EtOH (ALD) at 12-hour intervals. Lipid formation and changes were assessed by fluorescently labeling lipids with a 120 µl intravenous injection of SF44 dye (1.67 mM; SPARK Biopharma, South Korea) 37, 30 minutes before imaging. The SF44 dye features an absorption wavelength of 445 nm and an emission wavelength of 611 nm. Two hours after the last dose, mice were anesthetized and prepared for imaging. An imaging session was then conducted to assess alcohol-induced lipid accumulation and mitochondrial alteration.

## 3. Results

**Figure 1:**
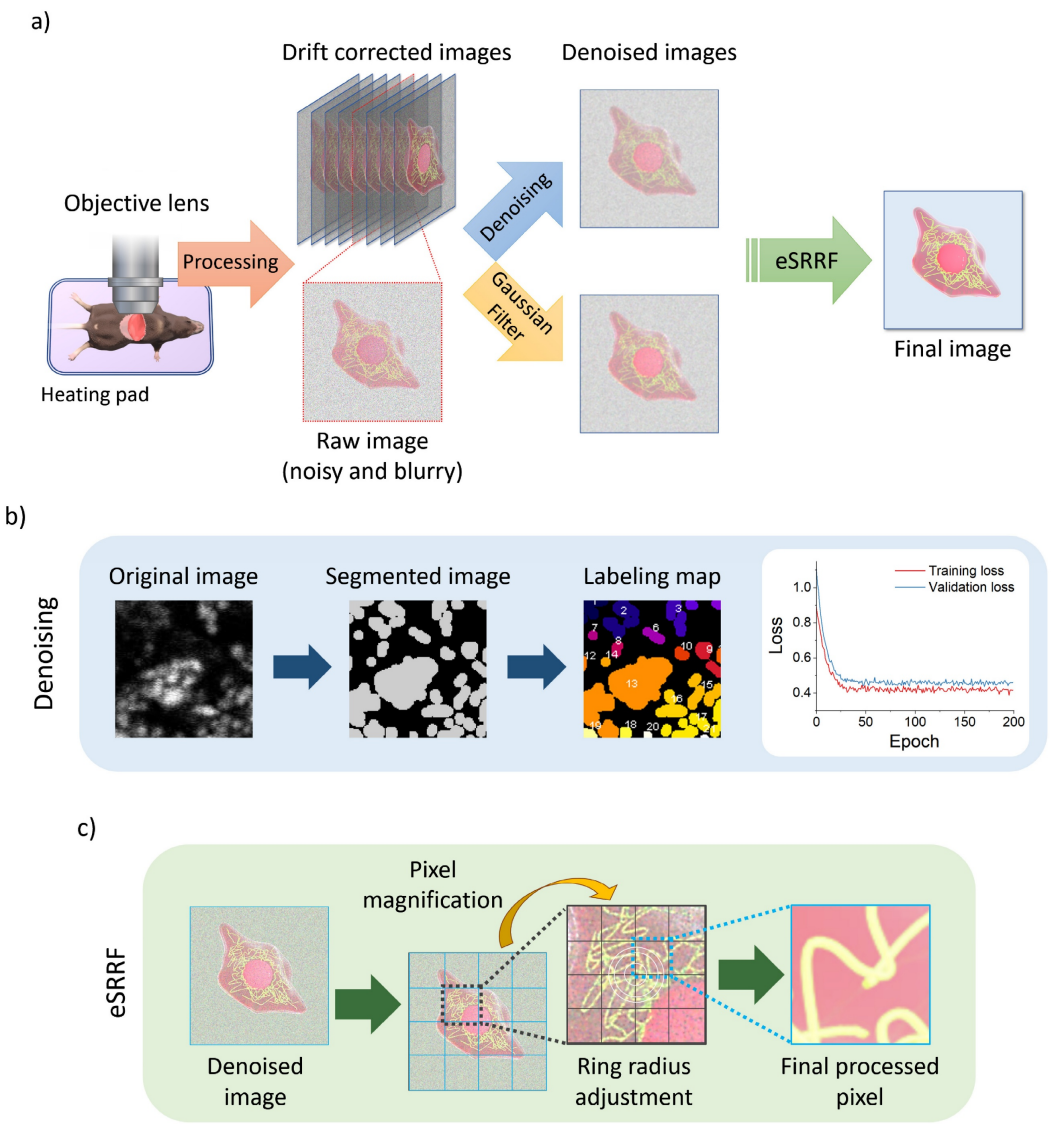
Intravital imaging workflow using two-photon microscope. a) Schematic of the intravital imaging procedure and subsequent image processing sequence, including raw data acquisition, stabilization, and preprocessing steps. b) Steps of image segmentation and labeling used in the denoising model, along with comparing training and validation loss plot. c) Sequence of the enhanced Super-Resolution Radial Fluctuations (eSRRF) algorithm, showing how the algorithm processes denoised image to achieve resolution enhancement and generate high-fidelity super-resolved reconstructions.

In this study, we aimed to achieve super-resolution imaging of subcellular structures *in vivo* by combining various numerical techniques. As shown in Figure 1, drift correction was performed after imaging to address potential motion artifacts. Subsequently, two different methods were applied and compared to optimize background noise reduction. The first method involved using a Gaussian filter to reduce any remaining background noise, while the second employed a trained denoising algorithm (Figure 1b) to enhance image quality by removing noise. Finally, the eSRRF algorithm (Figure 1c) was applied to both datasets to complete the image enhancement workflow.

### 3.1. Optimizing the eSRRF analyzing parameter

**Figure 2:**
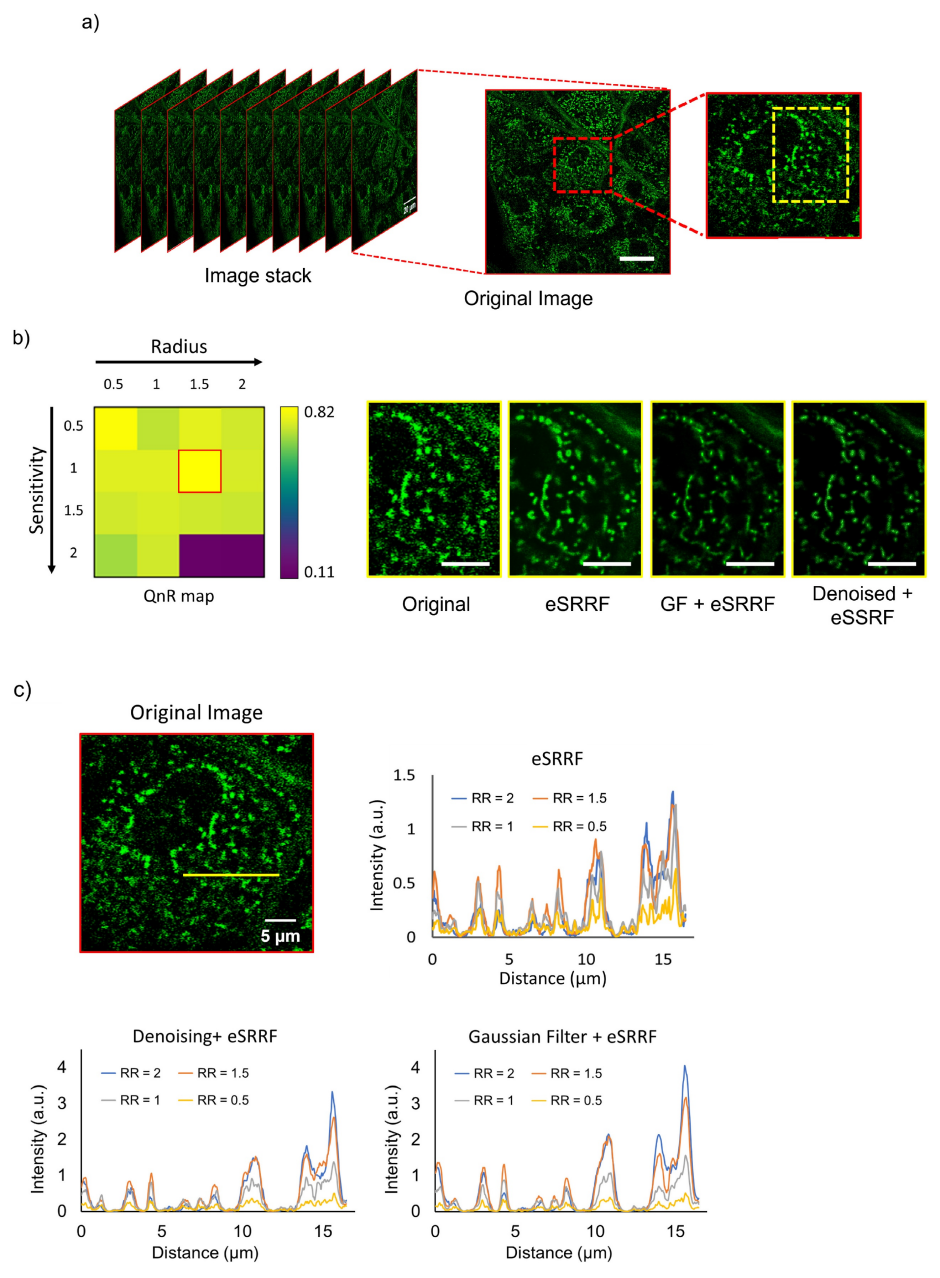
Comparison of skin imaging results with various image processing methods. a) Original image stack acquired from abdominal skin, with the inset on the right magnified to reveal finer details. b) The yellow inset profile from (a) was processed using various techniques, with RR and sensitivity values of 1.5 and 1, respectively, extracted from the QnR map. c) Intensity profiles along the line for various processed images and RR values. (RR: ring radius). Scale bars: (a) 20 µm (inset, 10 µm); (b) 5 µm.

To evaluate the effect of parameters employed in eSRRF processing on resolution enhancement, we imaged the dermal tissue of the abdominal skin, as shown in Figure 2a. The visual comparison clearly demonstrates the limitations of images captured without correction, characterized by blurriness and a lack of clear and detailed content. On the other hand, images obtained using the proposed method exhibit remarkable clarity, offering complete reproducibility during subsequent acquisitions. Using eSRRF, we systematically adjusted key parameters such as RR and sensitivity based on the best value in QnR map 17, utilizing the options available in the eSRRF plugin. This evaluation aimed to determine their effect on the level of detail in the final image. Based on the QnR score, the optimal RR and sensitivity values were identified as 1.5 and 1, respectively, as applied in original image and depicted in Figure 2b, these values are based on QnR map which shows the trade-off between fidelity and rFRC resolution. To quantitatively assess the enhancement achieved at each RR value, pixel intensity profiles were measured along the indicated line in the zoomed-in image from Figure 2a and its corresponding processed versions (Figure 2c). The intensity profiles revealed higher intensities for larger RR values, corroborating the qualitative improvement in the visualization of structures and features.

### 3.2. rFRC mapping

**Figure 3:**
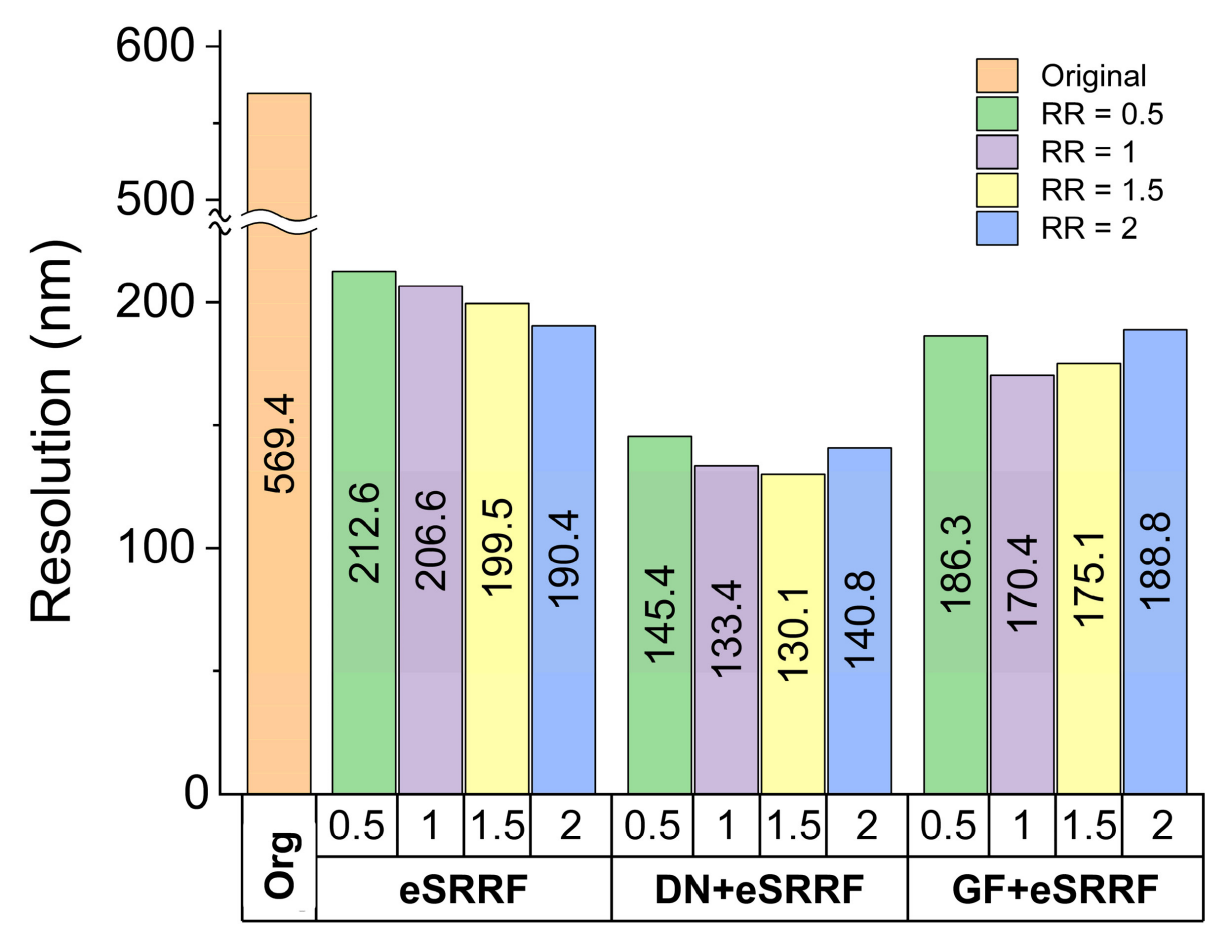
Comparison of image resolution obtained using different computational methods. Resolution was quantified using rFRC analysis, highlighting the improvement in spatial detail achieved by each approach. (Org: original, DN: denoising, GF: Gaussian filter, RR: ring radius).

The enhancement effect of increasing RR values on image resolution was further validated using rFRC analysis. As shown in Figure 3, the mean rFRC plot demonstrates a resolution improvement down to 130 nm achieved by combining the trained denoising model with eSRRF processing at the optimal RR of 1.5. It is important to note that while higher RR values can improve resolution, they may also result in the loss of some subtle features and introduce potential distortion in the final image. Therefore, when determining the optimal ring radius value, tradeoffs between preserving the features and enhancing finer resolutions have to be considered.

### 3.3. Epidermis image analysis

**Figure 4:**
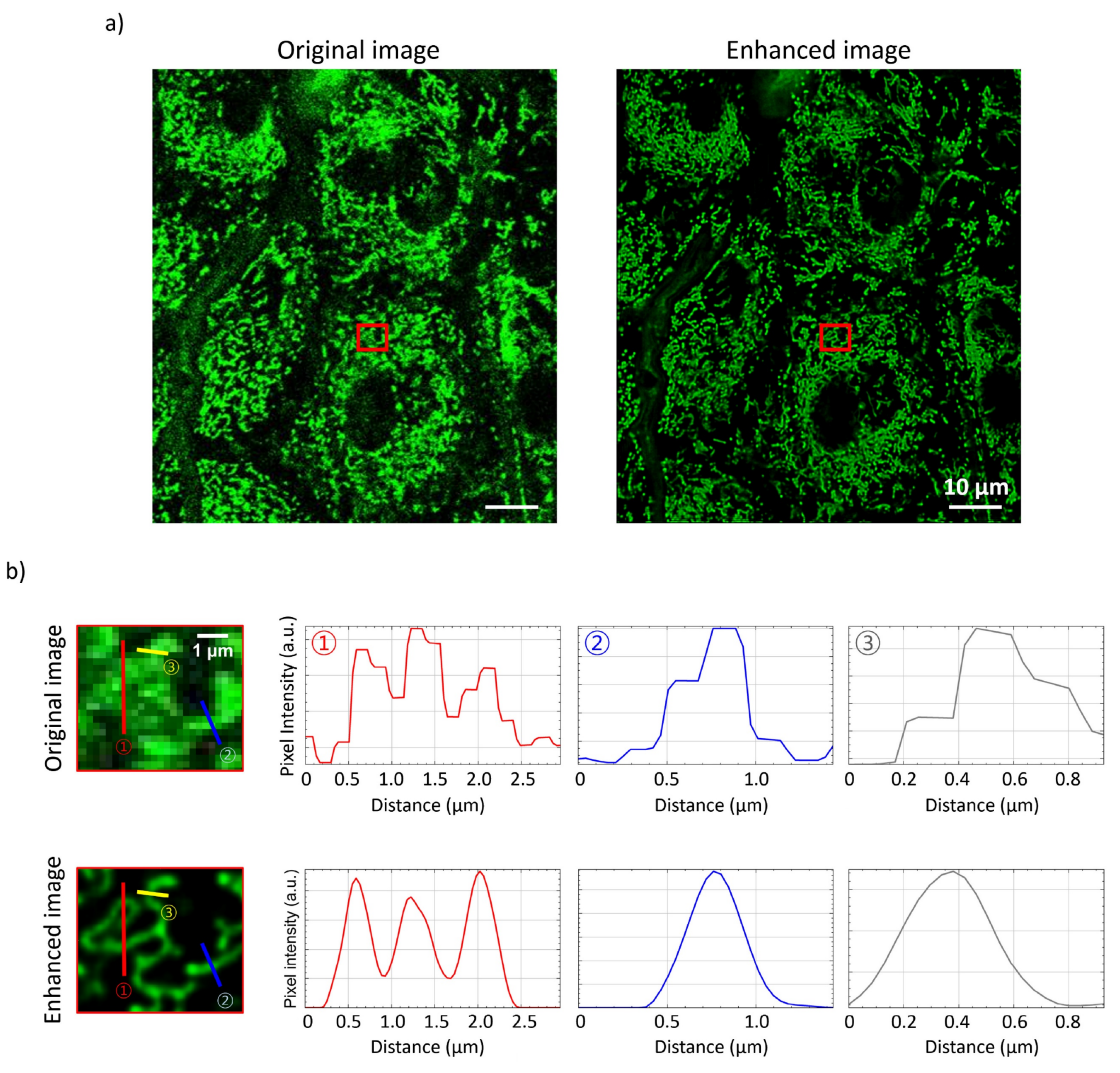
Epidermis mitochondrial images before and after processing. a) Original raw image of mitochondria and the corresponding processed image using the optimized parameters, highlighting improvements in clarity and resolution. b) Magnified regions showing detailed mitochondrial structures, accompanied by pixel intensity plots along the drawn line profiles in the zoomed-in images.

Following parameter optimization, the method was applied to the epidermis, where mitochondrial morphology was resolved and individual mitochondria were analyzed through pixel-intensity profiles in the original and enhanced images. Figure 4a displays a wide-field view of murine epidermal cells, while Figure 4b provides zoomed-in regions with pixel intensity plots along drawn profiles. Comparing these plots can reveal variations in curvature and intensity, highlighting various mitochondrial states and characteristics.

These findings demonstrate the potential of our methodology to advance understanding of mitochondrial behavior on the mechanisms underlying skin health and disease.

### 3.4. Visualization of lipid droplet accumulation in hepatocytes

**Figure 5:**
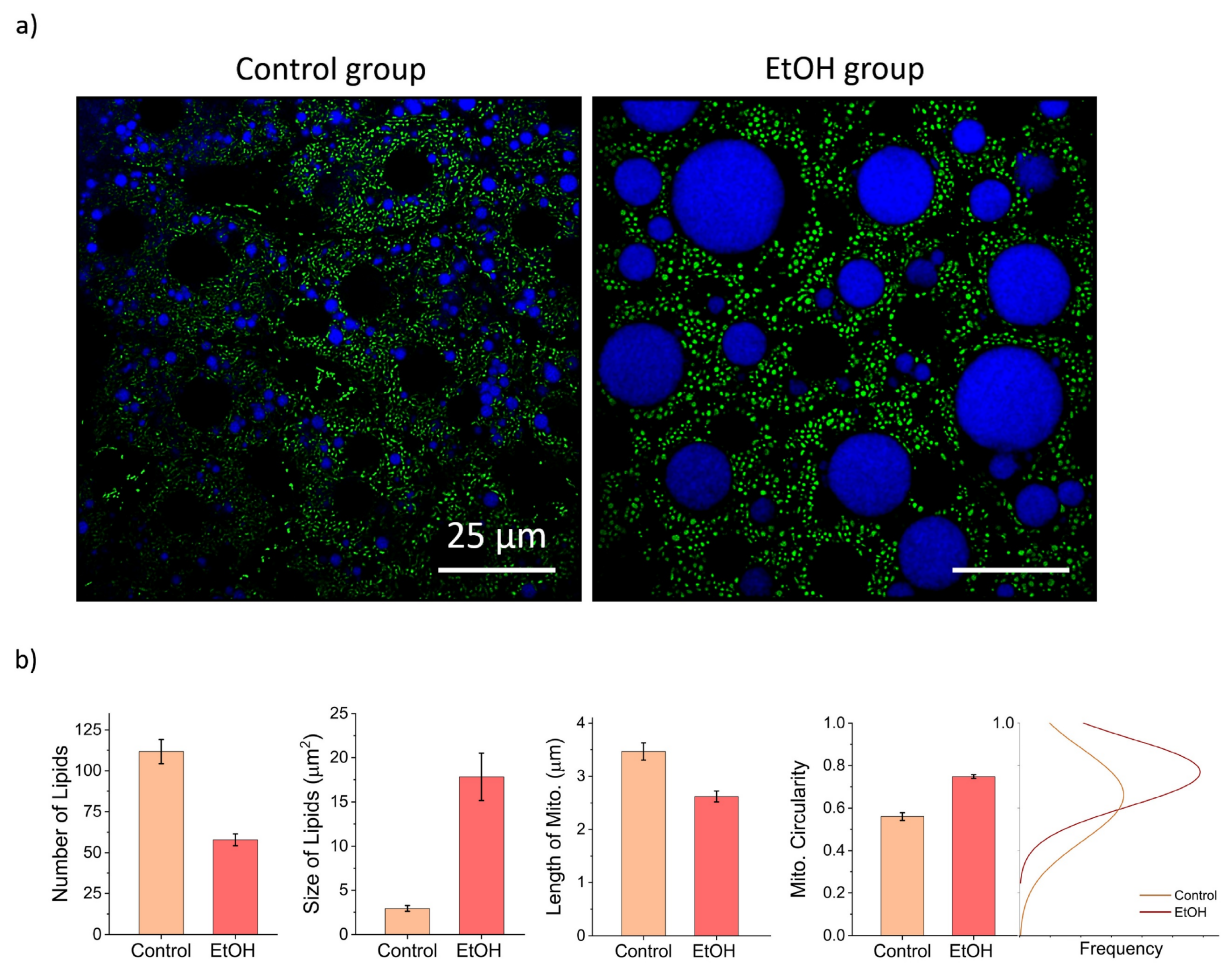
Imaging mitochondria and lipid droplets in liver. a) Representative images showing lipid droplets labeled with SF44 and mitochondria in the liver of control and EtOH-treated groups. Noticeable differences in the size, distribution, and number of lipid droplets, as well as alterations in mitochondrial morphology, are evident between the two groups. b) Quantitative comparison plots of lipid droplet size and number, along with mitochondrial morphological parameters, highlighting the structural changes induced by EtOH exposure. Scale bar, 25 µm.

To demonstrate the method's utility for pathological studies, we assessed lipid droplet accumulation in hepatocytes, with and without EtOH administration. Mice received intravenous injection of SF44 dye to fluorescently label lipids. Figure 5a shows pronounced lipid droplet formation in hepatocytes following EtOH treatment, consistent with alcohol-induced disruption of lipid metabolism. Specifically, EtOH is metabolized into acetaldehyde, which impairs fatty acid breakdown and promotes triglyceride accumulation in hepatocytes. Alcohol consumption also induces oxidative stress and inflammation in hepatic tissues, further aggravating lipid metabolism disruption and lipid accumulation. Figure 5b presents a comparative analysis of lipid vesicle size and quantity between control group on a standard diet and after EtOH treatment. The control group exhibited a higher number of smaller lipid droplets, whereas the EtOH-treated showed fewer but larger lipid droplets, highlighting EtOH's significant impact on lipid vesicle morphology.

Furthermore, Mitochondrial morphology was analyzed using the Mitochondrial Network Analysis workflow 38 in Fiji which quantifies parameters such as length and circularity of mitochondria. In the control group, mitochondria predominantly exhibited elongated, tubular morphologies, which are generally associated with efficient oxidative phosphorylation and healthy mitochondrial networks. In contrast, mitochondria in EtOH-fed group appeared more circular and fragmented, indicative of enhanced mitochondrial fission events. These morphological changes indicate ethanol-induced mitochondrial dysfunction, compromising energy production and increasing susceptibility to oxidative stress.

### 3.5. Visualization of other organs

**Figure 6:**
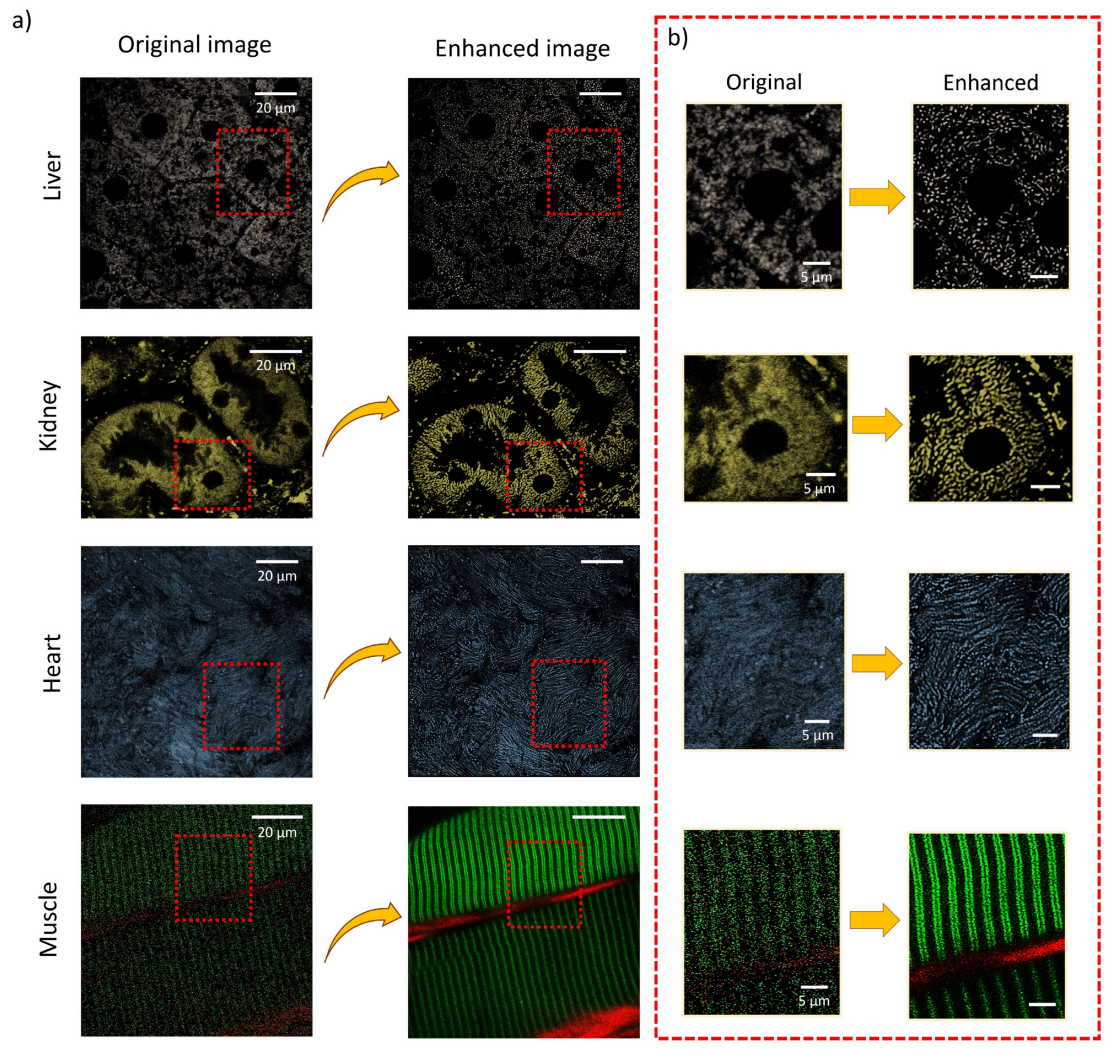
Images of various internal murine organs. a) Original and processed images of liver, kidney, heart, and skeletal muscle, illustrating the applicability of the imaging approach across multiple organs. b) Magnified regions showcasing fine subcellular details and the resolution improvements achieved through the enhancement pipeline.

Using insights from the pixel intensity plots, denoising and filtering strategies described above, we optimized the processing parameters. Subsequently, the internal organs, including the liver, kidney, heart, and muscle, which were gently prepared for intravital imaging. Figure 6 demonstrates images of these organs before and after processing using our proposed method, highlighting mitochondrial networks. Zoomed-in images reveal a notable improvement, where processed images showing significantly enhanced mitochondrial details and distinct cellular structures compared to the original ones. These results highlight the effectiveness of our method in capturing the intricate complexities of internal organs in a live animal.

## 4. Discussion

In this study, we introduced a computational approach to obtain detailed organelle imaging using two-photon microscopy in live animal studies, without the needs for specialized instruments, hardware modifications to the existing microscope setup, or additional costs. This makes the method broadly applicable and accessible for investigating organelle structure and function *in vivo*.

Two-photon microscopy offers several intrinsic advantages for intravital imaging, including deep tissue penetration, reduced photodamage, and localized excitation that minimizes out-of-focus fluorescence. Integrating multiphoton intravital microscopy and advanced image reconstruction algorithms, we can achieve super-resolution imaging *in vivo* that surpasses conventional limitations to visualize organelle structures in their native physiological environment.

Our method involves a two-step process: first, denoising using a trained model or Gaussian filtering to mitigate noise artifacts, followed by applying the eSRRF algorithm to enhance feature clarity in image.

Noise in two-photon and fluorescent imaging poses a significant challenge 39, stemming from the nature of photon emission and detection processes, amplified by device-specific electronic and optical noise sources. Self-supervised denoising algorithms address this issue by adapting to these unique noise patterns through iterative learning on both single and multiple image datasets.

To optimize the parameters involved in the eSRRF processing, parameters sweep was performed. For instance, the radiality ratio (RR) was optimized to a value of 1.5 and sensitivity to 1, based on computational results. This configuration enabled a substantial improvement in resolution, as quantified by rRFC analysis, enhancing the original image resolution from 570 nm to 130 nm in the processed image.

To validate our method, we performed control experiments using conventional microscopy techniques. Results consistently showed that our method outperformed traditional approaches in resolution, contrast, and fidelity. Its robust performance across various sample types, including cellular and tissue specimens, underscores its versatility and potential for broader applications. Preliminary experiments further affirmed its effectiveness, with improved resolution and enhanced features demonstrating its value in advancing imaging techniques for animal studies.

To investigate a clinical relevance, we applied our method to a fatty liver disease model induced by EtOH administration. Observed changes in lipid vesicles and mitochondria, suggest that EtOH affects lipid metabolism and mitochondrial monitoring, leading to morphological alterations. Increased lipid droplet area in EtOH-treated samples may indicate lipid accumulation 40 or changes in membrane fluidity, potentially affecting cellular function. The shift from tubular to circular mitochondrial morphology points to EtOH-induced mitochondrial fission, which could impact energy production and biomaterial metabolism. These findings highlight the need for further research into the molecular mechanisms underlying these changes.

Despite its effectiveness in our study, further research is needed to evaluate the applicability of this method across diverse physiological and pathological conditions. This is particularly important to address the inherent technical limitations of intravital imaging, such as tissue motion artifacts induced by physiological activity (e.g., breathing, heartbeat), limited signal-to-noise ratio due to weak fluorescence signals, and light scattering that compromises resolution in deeper tissue layers. In addition, the computational processing remains another challenge, as training the denoising model and performing eSRRF analysis can require several minutes for large datasets. Optimization of these parameters for varying experimental conditions will therefore be essential to broaden the method's applicability.

Successful application in live animal studies could support clinical translation, enhancing diagnostics and treatment monitoring. Future work will focus on adapting the method to various physiological and pathological conditions to uncover new biological insights and advances in vivo imaging. Furthermore, expanding the technique beyond two-photon microscopy to integrate with other imaging modalities holds the potential to further enhance human imaging capabilities and deepen our understanding of disease mechanisms at both cellular and systemic levels.

## 5. Conclusion

The proposed computational approach offers a cost-effective and accessible solution for achieving super-resolution imaging *in vivo* without the need for high-end microscopy equipment. By combining two-photon microscopy with a trained denoising model and eSRRF, we significantly enhanced images resolution, enabling detailed visualization and analysis of subcellular while minimizing phototoxicity, an essential advantage for long-term live-animal imaging. Specifically, our technique allowed in vivo analysis of mitochondria under both physiological and alcohol-induced stress conditions, providing new insights into mitochondrial alterations associated with ALD. Additionally, this technique advances *in vivo* imaging across various biological contexts, supporting research into intracellular trafficking, organelle dynamics, and cellular stress responses within their native environments. Future work will focus on refining the image processing pipeline and adapting the approach to additional imaging platforms and experimental models in living organism studies.
